# Digitalization of Comprehensive Geriatric Assessments for Nursing Practice: A Feasibility and Proof-of-Concept Study Toward Nursing Home Implementation

**DOI:** 10.3390/healthcare14040528

**Published:** 2026-02-19

**Authors:** Uijin Park, Midori Miyagi, Xinze Wu, Makoto Ito, Manabu Chikai, Fuminori Sakai, Tomofumi Miura, Hiroshi Sato, Akihiko Murai, Shannon Freeman, Satoru Ebihara

**Affiliations:** 1 Department of Rehabilitation Medicine, Tohoku University Graduate School of Medicine, Sendai 9800873, Japan; park.uijin.q5@dc.tohoku.ac.jp (U.P.); midori.miyagi.b5@tohoku.ac.jp (M.M.); wu.xinze.r3@dc.tohoku.ac.jp (X.W.); 2 ISB Corporation Business Headquarters, R&D Promotion Office, Minato-ku 1080075, Japan; ma_ito@isb.co.jp; 3 National Institute of Advanced Industrial Science and Technology, Tsukuba 3058560, Japan; m-chikai@aist.go.jp (M.C.); sato.hiro@aist.go.jp (H.S.); a.murai@aist.go.jp (A.M.); 4 Sakura Tech Corporation, Kanagawa 2220033, Japan; sakai@sakuratech.jp; 5 National Cancer Center Hospital East, Kashiwa 2778577, Japan; tomiura@east.ncc.go.jp; 6 School of Nursing, University of Northern British Columbia, Prince George, BC V2N 4Z9, Canada; shannon.freeman@unbc.ca

**Keywords:** geriatric nursing, health assessment, wearable electronic devices, environmental monitoring, sensors, quantified self

## Abstract

**Background/Objectives**: Comprehensive Geriatric Assessment (CGA) is essential for maintaining quality of life (QOL) and independence in older adults. Still, its implementation is labor-intensive and difficult to sustain in aging societies such as Japan. Digital technologies may enable continuous, scalable CGA in daily living environments. This study aimed to develop and preliminarily evaluate a digital CGA (D-CGA) framework by integrating data from multiple monitoring devices, as a preparatory step toward Artificial Intelligence (AI)-supported personalized care planning. **Methods**: Four devices (Handy, Apple Watch, Withings Sleep, and Vieureka) were selected. Due to ethical constraints in Japan, a pilot study was conducted with graduate students. Participants underwent continuous monitoring for five weekdays. Common and device-specific measurement items were extracted, visualized, and compared across devices. Heart rate data were examined using correlation-based analyses. Baseline CGA was conducted before monitoring. **Results**: Distributional and temporal characteristics of physiological measures were explored separately for daytime and nocturnal periods. Continuous heart rate and respiratory rate data were successfully collected across monitoring days, demonstrating the feasibility of real-life data acquisition using the selected devices. Heart and respiratory rates showed distinct distributional patterns between daytime and nocturnal periods, supporting context-specific physiological characterization. **Conclusions**: This pilot study demonstrates the feasibility of integrating multi-device data for D-CGA and provides foundational reference data for future studies of older adults. The results support the potential of D-CGA to inform personalized care and guide subsequent large-scale and clinical investigations.

## 1. Introduction

With advances in science and technology, population aging has become an increasingly serious issue, and the health of older adults is a primary social concern [1]. Although medical progress and the expansion of healthcare resources have extended average life expectancy, chronic diseases, cognitive decline, and reduced ability to perform activities of daily living are becoming more common. Improving QOL for older people is now a critical challenge [2]. Japan now faces a severe demographic crisis [3]. The total fertility rate dropped to 1.2 in 2023, and the population is rapidly aging [3]. While the older adult population continues to grow, the working-age population supporting them is steadily declining [3]. This demographic imbalance poses significant challenges to healthcare and social welfare systems.

In Japan’s long-term care insurance system, care services for older adults are classified into several categories, including in-home (home-based) services, facility (institutional) services, community-based services, and care management services [4] (Table 1). Home-based care enables older adults to remain in their own homes while receiving support such as visits from care workers or nurses, and access to day centers. Care management involves care managers who assess each individual’s situation and create tailored care plans to coordinate appropriate services. Institutional care refers to residential facilities that provide 24 h support, including public nursing homes for those requiring high levels of care, rehabilitation-focused senior health service facilities, privately operated paid care homes, and small-scale group homes for people with dementia. Medical facilities include hospitals and clinics that provide inpatient and outpatient care depending on the older people’s medical needs. These care options are designed to provide appropriate support based on each senior’s health condition and living environment. However, due to high demand, many facilities have long waiting lists [5,6].

CGA is a useful resource to evaluate the health status of older adults from multiple perspectives, including physical, mental, psychological, social, and environmental aspects. The primary purpose of CGA is to create individualized care plans based on assessment results, aiming to improve the QOL and independence of older adults [3].

This study helps to inform a system that could gather sufficient data for AI to generate efficient, accurate care plans, automating and digitizing CGA within older people’s households. First, a six-month investigation was conducted on suitable monitoring devices and AI technologies. Four types of monitors were selected based on the accuracy of measuring physiological information and vital signs, as well as ease of operation. The goal is to simplify CGA implementation using digital devices and clearly visualize the results. CGA should enable evidence-based development of individualized care plans. A total of 13 device types were evaluated for use in real-life use cases for older people. Consideration factors included: Discomfort or resistance during wear, and whether advanced technical skills were required. For example, ring-type devices are highly functional, but issues may arise if the size is inappropriate. There is also a risk that they come off during handwashing or go unnoticed. Wristwatch-type devices were also tested, with an Apple Watch demonstrating excellent performance. Based on these factors, four devices were ultimately selected that combined practical ease of use with accurate data measurement.

Facility-based experimental research in Japan requires strict ethical review. Because the monitoring devices used in this study had not previously been applied in clinical settings, preliminary validation of their ability to detect human movement was necessary. In addition, given the potential privacy concerns associated with camera-based monitoring, a pretest was conducted to assess possible psychological burden among participants. Therefore, prior to implementing these devices in residential care facilities, it was essential to evaluate their feasibility. As an initial step, measurements were conducted using the four selected devices with graduate students as participants.

## 2. Materials and Methods

### 2.1. Subjects

**Figure 1:**
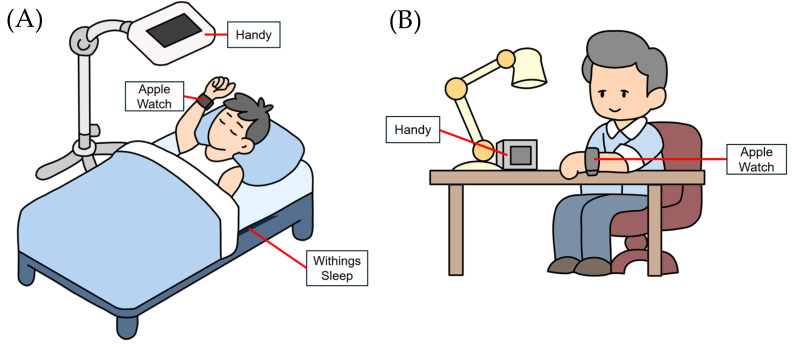
Schematic Diagram of Measurement Method. (**A**) Measurement during sleep. (**B**) Measurement during daytime.

**Figure 2:**
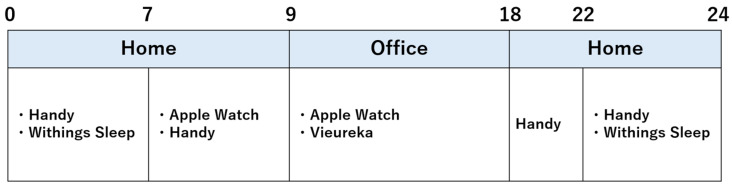
Time Schedule.

This study used four devices with healthy adults: Apple Watch (Apple Inc., Cupertino, CA, USA), Withings Sleep (Withings, Issy-les-Moulineaux, France), miRadar^Ⓡ^8 Handy (Sakura Tech., Kanagawa, Japan), and Vieureka (Vieureka Inc., Osaka, Japan) [7,8,9,10]. An Apple Watch and a Withings Sleep are contact-type devices, while a Handy and a Vieureka are non-contact devices that require installation at home and in the office, respectively. During sleep, a Handy and a Withings Sleep were used simultaneously to measure sleep conditions (Figure 1). Daily activities at home were calculated using a Handy and an Apple Watch (Figure 1). In the office, measurements were taken using a Vieureka and an Apple Watch. Recruitment was conducted via posters posted at Tohoku University Graduate School of Medicine. A total of five students applied: two males and three females. Each student was monitored for five business days (Figure 2).

### 2.2. Monitor Specifications—Contact Type

Two contact-type devices were selected. The first is the Apple Watch, worn on the wrist. It can measure heart rate, detect a history of atrial fibrillation (AFib), and perform electrocardiogram (ECG) measurements. ECGs recorded by an Apple Watch can produce waveforms like those of an I-lead ECG. Availability varies by country, but it is currently approved for use in Japan. An electrical heart sensor records heart rate and rhythm, aiding in the detection of atrial fibrillation (AFib), a common arrhythmia. It also allows graphical display of electrical signals. Furthermore, it can track a wide range of biometric and activity data, including: Cardiovascular notifications, Respiratory rate, Activity level (time standing, resting energy expenditure, walking/running distance, step count, physical activity score, activity energy, exercise time, walking stability, stair climbing count/speed, walking speed, walking asymmetry, double support time, stride length, ambient noise level, fall detection), Blood oxygen saturation (SpO_2_), Sunlight exposure, Sleep Parameters (Sleep Duration, Awakening Count, REM Sleep, Deep Sleep, Respiratory Rate, Heart Rate, Average Wrist Temperature, etc.). The second device is Withings Sleep. This device is placed under the mattress to measure sleep duration, sleep state, sleep rhythm, and snoring, generating a comprehensive sleep score. We aim to use this device to monitor the sleep patterns in older adults who are uncomfortable wearing wearable devices.

### 2.3. Monitor Specifications—Contactless Type

Two contactless-type devices were selected. The first contactless monitoring device is a Handy, provided by our research collaborator at Sakura Tech.

Handy is a non-contact vital-signs monitoring system based on a 24-GHz-band Multi-Input Multi-Output (MIMO) frequency-modulated continuous-wave (FMCW) radar architecture. The system emits radio-frequency signals that can penetrate common indoor obstructions, such as clothing, bedding, and wooden doors. Because radar sensing is independent of ambient illumination, continuous monitoring can be performed regardless of lighting conditions and without requiring a direct line of sight. Furthermore, as the system does not capture optical images or biometric identifiers, it does not enable personal identification, thereby preserving user privacy.

Distance measurement is achieved using FMCW radar principles, in which range is estimated from the beat frequency between transmitted and received signals. A MIMO antenna array enables electronic beamforming, allowing angular estimation of targets. By combining range and angular information, the system localizes subjects within a two-dimensional spatial grid.

The maximum detectable range is approximately 5 m, with a horizontal field of view of ±40° and a vertical field of view of ±8°. For vital sign extraction, radar signal processing techniques are applied to isolate reflections originating from the localized subject position. The received signals contain components corresponding to gross body motion as well as micro-displacements caused by respiration and cardiac activity. Since cardiac-induced surface displacements are significantly smaller in amplitude than voluntary or involuntary body movements, frequency-domain filtering is applied to separate physiological signals from large-scale motion artifacts. Heartbeat-related components are continuously quantified, and transient outliers are suppressed through temporal comparison with preceding and subsequent measurements. The system samples data at 0.5 s intervals. Acquired data can be stored locally on a USB storage device or transmitted to a cloud server via Wi-Fi connectivity. The device operates in compliance with Japan’s Radio Law and is approved for general public use. Regulatory approval in Canada is currently under consideration.

The second contactless device is Vieureka, which is an AI camera that uses “pose estimation AI” operated internally to reconstruct the 3D motion of subjects. Pose estimation is a technology that estimates positions in a camera’s 2D coordinate, corresponding to joints such as the “nose, shoulders, elbows, and knees” of a person when they appear in the input image. For 3D reconstruction, we measured 3D coordinates of 4 Viurekas in advance and synchronized the shooting timing using NTP. Then it becomes possible to measure a subject’s 2D coordinates to reconstruct the 3D position of the subject at a given time. Since Viureka has a LAN interface, it can also transmit this data to servers or other systems via IP. Using periodic data notifications from each Viureka, 3D motion (time-series information of 3D keypoint coordinates) can be generated using reconstruction technology owned by ISB Corporation (patent pending). In this study, we aimed to measure individual walking speed, posture, and sit-to-stand transition speed using four of these devices.

### 2.4. D-CGA Data Collection

Data from the participants were anonymized prior to being analyzed statistically. Prior to the initiation of monitoring, all participants underwent a CGA assessment. The CGA assessment focused on ADL (Activities of Daily Living), cognitive function, motivation, and indicators of depression. The Barthel Index [11], Scale of Lawton [12], and Vulnerable Elders Survey-13 (VES-13) [13] were used as ADL indicators; the MMSE [14] as a cognitive function indicator; the Vitality Index [15] as a motivation indicator; and the Geriatric Depression Scale-15 (GDS-15) [16] as a depression indicator. Four types of monitors were used 24 h a day, five days a week, to measure key activities and sleep (Figure 2). All data preprocessing, descriptive analyses, and visualizations were performed using custom Python scripts (version 3.13).

## 3. Results

### 3.1. CGA Results in Healthy Participants

Since all subjects were healthy individuals, none exhibited problems with the CGA before or after the actual measurement.

### 3.2. Data Trends Across Three Devices

For an Apple Watch, a Withings Sleep, and a Handy, trends for each item could be confirmed. These results showed that they can serve as reference values in D-CGA. The list of acquired data for the three devices is as follows (Table 2).

**Figure 3:**
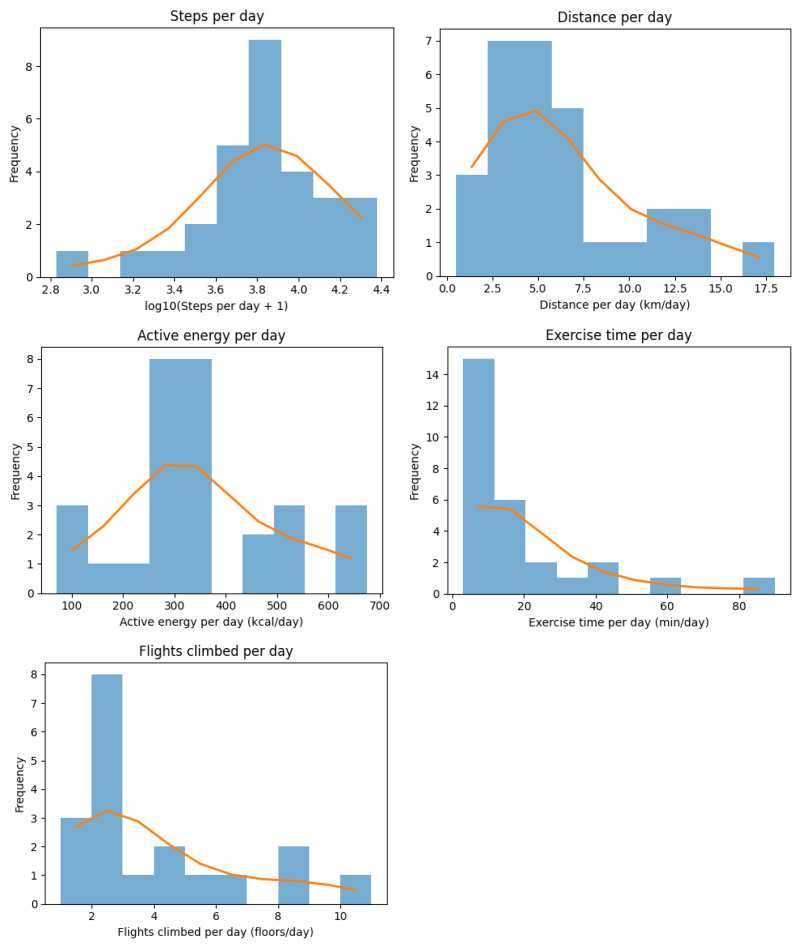
Activity-related results, as measured by Apple Watch.

Step count, walking distance, active energy expenditure, exercise time, and stair climbing measured by the Apple Watch showed the same trend among all participants (Figure 3). Histograms with kernel density estimates were used to visualize the distribution of the variables.

**Figure 4:**
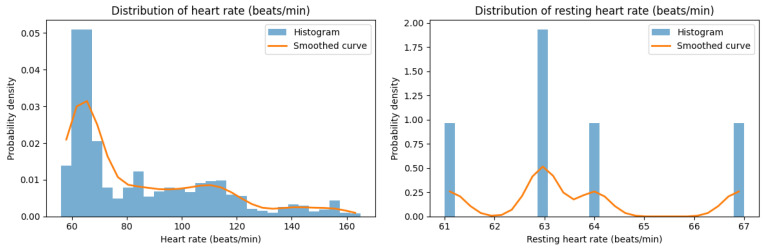

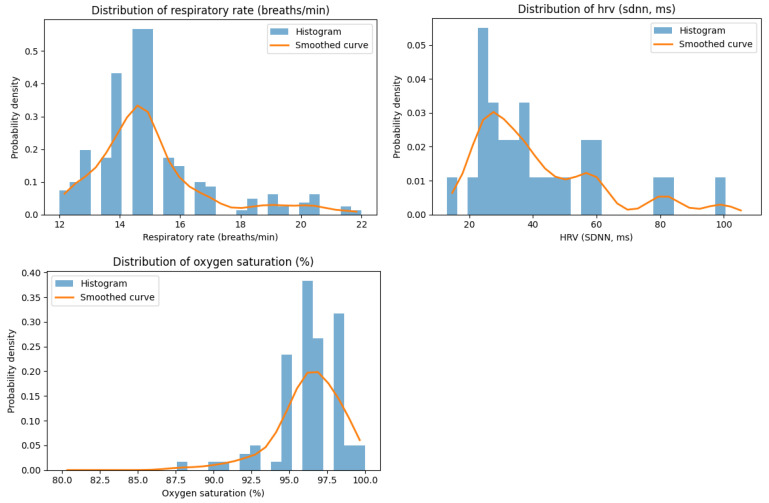
Sleep analysis, as measured by Withings Sleep.

Next, an issue in Withings Sleep data occurred: the Sleep Heart Rate Distribution item could be measured for only two individuals, while Resting Heart Rate, Respiratory Rate, Heart Rate Variability, and Oxygen Saturation could be calculated for only one (Figure 4). Factors contributing to this variability may include turning over during sleep, bed height, mattress thickness, and room size. Subjects for whom measurements were feasible in this study all resided in dwellings with two or more rooms, whereas subjects for whom measurement proved difficult were living in studio apartments. Standardizing the experimental environment is a minimum requirement for ensuring measurement reproducibility. However, the ultimate goal of this study is to remotely observe the condition of individuals living in their home environment. It is unrealistic to make all living environments identical. Therefore, a key challenge for this research in the future is to quantitatively clarify the extent to which data can be obtained under different environmental living conditions and the impact environmental factors have on measurement results.

**Figure 5:**
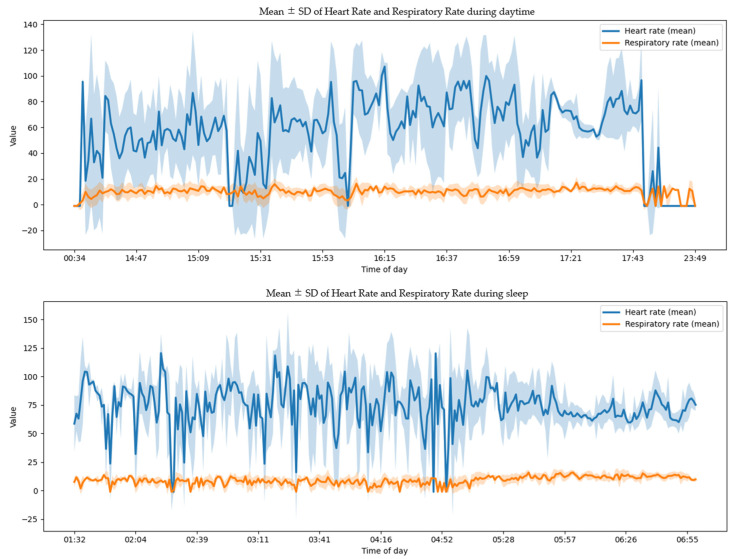
Heart rate and respiratory rate, as measured by Handy.

Data from Handy were analyzed as an exploratory time series using 1 min binned means and standard deviations. Physiological signals were averaged within 1 min epochs to characterize nocturnal trends (Figure 5).

The valid data ratio for heart rate and respiratory rate during desk measurements was consistently high across participants, indicating stable data acquisition under free-living conditions. It conducted an analysis evaluating data quality and the feasibility of continuous biological data using the effective data rate metric (Table 3). Data quality was assessed using a rule-based validation approach, in which only data segments meeting predefined physiological plausibility criteria for heart rate (HR) and respiratory rate (RR) were considered valid (HR: 30–220 bpm; RR: 5–40 breaths/min). The valid data ratio was calculated as the proportion of valid data points among all recorded measurements for each participant (valid points/total points × 100; Table 3).

**Table 3:** The valid data ratio for heart rate and respiratory rate during desk measurements.

Subject	Total Points	Valid HR (%)	Valid RR (%)
1	31,902	100.0	96.71494
2	37,702	100.0	100.0
3	232,972	100.0	100.0
4	282,208	100.0	100.0
5	111,295	100.0	100.0
Mean ± SD		100.0 ± 0.0	99.3 ± 1.5

**Abbreviations:** HR, heart rate; RR, respiratory rate.

### 3.3. Data Variation (Table 4)

Data acquisition variability was observed across three devices: the Apple Watch, Withings Sleep, and Handy. First, it was necessary to determine whether this variability stemmed from physiological fluctuations or measurement noise.

Physiological variability may help detect abnormalities. Furthermore, significant variability in unacceptable parameters may indicate measurement errors or disease.

It is necessary to discern meaningful fluctuations within variability and redesign for applicability to patients.

**Table 4:** Data variation.

Acceptable Variables (Physiological Variability)	Unacceptable Variables (Should Be Stable)
Heart rate variability, heart rate, activity level, energy expenditure, etc. Fluctuations due to hormonal balance Significant changes during illness	Oxygen saturation, resting heart rate, and sleep stages

### 3.4. Demonstrable Plan for Patient Application

This study recognized the necessity to identify meaningful variations within data variability and develop measurement and evaluation designs that can be applied to patients in the future. Although physiological variability was observed in the data obtained from healthy subjects, no unacceptable variables that would pose measurement problems were identified.

This variability in healthy individuals can be utilized as foundational data for establishing “normal value standards” in the future. On the other hand, standardizing the measurement environment is crucial to minimizing variability. Specifically, environmental factors such as room size, bed height, and mattress thickness were suggested to potentially influence data variability. Therefore, when conducting future studies in nursing homes, it is necessary to clarify beforehand which variations arise from differences in environmental conditions before performing measurements. The future goal of this research is to establish clinically applicable reference ranges for normal values based on these findings.

## 4. Discussion

Conventional CGA has traditionally been conducted through in-person, multidisciplinary evaluations in clinical settings and has been widely recognized as a valuable tool for assessing functional status, cognition, and overall health in older adults [17]. However, these assessments are often time-consuming and resource-intensive, which may limit their scalability in rapidly aging populations. In light of these challenges, the present study sought to explore the feasibility of a digital approach to CGA.

Recent studies have explored the use of wearable devices and remote monitoring technologies for assessing physiological and functional parameters, including physical activity, sleep patterns, and cardiovascular indicators [18,19]. These approaches have demonstrated the potential of digital health technologies to provide continuous and objective health data outside clinical environments [20]. However, most previous studies have focused on single-domain monitoring rather than comprehensive, multidimensional assessment.

As a pilot study, we examined the feasibility of implementing a digital alternative to CGA, the core component of this research. Specifically, we investigated how shared CGA-related concepts, such as activity and sleep, are captured across multiple consumer-grade monitoring devices, conducting a pre-feasibility and proof-of-concept study prior to hospital implementation.

This work is situated within the growing literature on the *quantified self* and self-tracking, where continuous collection of personal health data has become increasingly accepted in daily life. Previous study suggests that individuals are more willing to collect and share self-generated health data with clinicians, recognizing its potential value for personalized assessment and decision-making [21].

By demonstrating the feasibility of multi-device, real-world physiological monitoring, this study supports the potential for integrating in-home self-tracking data into digital CGA frameworks. Such integration may enable the collection of higher-quality, context-rich data in everyday environments, ultimately contributing to more accurate assessment and improved, individualized care planning in clinical practice.

Establishing such baseline patterns in healthy participants is a critical step toward applying digital CGA to patient populations and advancing personalized medicine. Reference distributions derived from healthy individuals provide context for interpreting patient data, while metrics characterized by high variability, such as gait asymmetry, highlight the importance of standardized measurement conditions. At the same time, these variable indicators remain clinically valuable when interpreted longitudinally at the individual level.

Building on the demonstrated feasibility of real-world data collection, future opportunities include the development of monitoring algorithms that define personalized tolerance ranges and prioritize data quality over data quantity, for example, by emphasizing continuous and sufficiently frequent measurements. Such approaches may enable more reliable interpretation of in-home monitoring data and support more precise, individualized care planning.

This study has several limitations. First, it was designed as a pilot and feasibility study with a small sample size, limiting statistical inference and generalizability. Second, participants were healthy graduate students rather than older adults, and physiological and behavioral patterns may differ from those of the target population. Nevertheless, these limitations are appropriate for an exploratory study aimed at clarifying data structure, device complementarity, and the feasibility of D-CGA prior to large-scale implementation in older adult populations. The third relates to data synchronization across devices. Because the sampling frequencies differed among the measurement systems, precise synchronization of the collected data was challenging. This may have affected the temporal alignment of physiological and behavioral signals across devices. In future studies, particularly those involving patient populations, careful consideration of device selection and sampling characteristics will be necessary to improve data integration and synchronization.

## 5. Conclusions

The primary feasibility outcome of this study was the successful collection of baseline physiological and behavioral data necessary for the digitization of CGA and its future integration with AI-driven analysis. Through continuous monitoring, we identified the practical need to distinguish whether observed fluctuations reflected true physiological changes or measurement-related noise. Certain parameters, such as activity levels, heart rate variability, and energy expenditure, exhibited expected day-to-day variability influenced by factors including hormonal balance and transient health conditions. In contrast, metrics such as oxygen saturation, resting heart rate, and sleep stages were relatively stable under healthy conditions, suggesting that substantial deviations in these measures may indicate measurement artifacts or emerging health issues.

## Figures and Tables

**Table 1:** Long-Term Care Services for Older People in Japan and Their Categorization.

Type	Description	Examples/Features
Care Management	Care managers assess the situation and create/adjust care plans	Creating and adjusting personalized care plans
Home Care	The older people live at home while receiving care services	Home-visit care (helpers come to the home) Home-visit nursing (nurses come to the home) Day services (daycare facilities)
Facility Care	The older people live in facilities and receive care	Special nursing homes (for high-care needs) Geriatric health services facilities (focus on rehabilitation) Private nursing homes Group homes (small-scale homes for dementia patients)
Medical Institutions	Hospitals and clinics where older people receive medical treatment	Hospitals, clinics

**Table 2:** The list of acquired data for the three devices.

Measurement Metrics	Apple	Withings	Sakura Tech	Panasonic
Measurement Metrics	Apple Watch	Withings Sleep	Handy	Vieureka
Respiratory Rate	〇	〇	〇	―
SpO_2_	〇	―	―	―
Activity Level	〇	―	―	〇
Resting Heart Rate	〇	〇	〇	―
Heart Rate Variability	〇	―	―	―
Walking, Running	〇	―	―	〇
Step Count	〇	―	―	〇
Exercise Duration	〇	―	―	〇
Stair Climbing Speed	〇	―	―	―
Sleep Assessment	〇	〇	―	―
Sleep Duration	〇	〇	―	―
Sleep Heart Rate	〇	〇	〇	―

**Abbreviations:** SpO_2_, peripheral capillary oxygen saturation. 〇, available; ―, not available.

## Data Availability

The datasets generated and analyzed during the current study are not publicly available due to ethical restrictions (e.g., containing potentially identifiable subject information), but are available from the corresponding author upon reasonable request.

## Abbreviations

The following abbreviations are used in this manuscript:

AI	Artificial Intelligence
CGA	Comprehensive Geriatric Assessment
D-CGA	Digital Comprehensive Geriatric Assessment
GDS-15	Geriatric Depression Scale-15
HR	Heart rate
QOL	Quality of Life
RR	Respiratory rate
SpO_2_	Peripheral capillary oxygen saturation
VES-13	Vulnerable Elders Survey-13
